# Lithium Dispensing Patterns in Dutch Youth: Prevalence, Incidence Dosages, and Duration of Use From 2011 to 2022

**DOI:** 10.1111/bdi.70134

**Published:** 2026-06-17

**Authors:** Ravish N. Gangapersad, Lisa T. Ringeling, Jens H. J. Bos, Birgit C. P. Koch, Manon H. J. Hillegers, Pilar Garcia‐Gomez, Catharina C. M. Schuiling‐Veninga, Eelko Hak, Bram Dierckx

**Affiliations:** ^1^ Department of Hospital Pharmacy Erasmus University Medical Center Rotterdam the Netherlands; ^2^ Erasmus School of Economics Erasmus University Rotterdam Rotterdam the Netherlands; ^3^ Rotterdam Clinical Pharmacometrics Group Erasmus University Medical Center Rotterdam the Netherlands; ^4^ Department of Child and Adolescent Psychiatry/Psychology Erasmus University Medical Center Rotterdam the Netherlands; ^5^ Groningen Research Institute of Pharmacy (GRIP), Unit Pharmacotherapy, ‐Epidemiology and ‐Economics (PTEE) University of Groningen Groningen the Netherlands; ^6^ Department of Clinical Pharmacy and Pharmacology University of Groningen, University Medical Center Groningen Groningen the Netherlands

**Keywords:** dosages, duration of use, Dutch youth, incidence, lithium, prevalence, psychopharmacoepidemiology

## Abstract

**Objective:**

Bipolar disorder (BD) often emerges during mid‐adolescence and young adulthood, and leads to functional and social impairment, with a prevalence of 0.87% among Dutch youth. Lithium, the first‐line BD maintenance treatment, stabilizes mood, prevents suicide, and is well tolerated, yet its use has declined as long‐term second‐generation antipsychotics (SGAs) prescriptions increased. This study explores the prevalence, incidence, duration, prior and concurrent use of psychotropic medications, and dosage patterns of lithium dispensing in Dutch youth.

**Method:**

A retrospective cohort study using the Dutch community pharmacy‐based IADB.nl database included 567 youths (6–25 years), who were dispensed lithium between 2011 and 2022.

**Results:**

The yearly prevalence of lithium dispensing per 100,000 fluctuated between 30.3 in 2011 and 34.8 in 2022 (*p* = 0.40), with the lowest prevalence in 6–12‐year‐olds and highest in 20–25‐year‐olds. Females had overall higher prevalence rates than males (*p* = 0.03). Overall incidence rose from 7.9 to 9.3 (*p* = 0.68). In 2011, incidence was 10.7 for both the 13–19 and 20–25 age groups. By 2022, this decreased to 4.2 in 13–19‐year‐olds and increased to 19.0 in 20–25‐year‐olds. Approximately 55% of individuals received other psychotropics before lithium, and 52% concurrently, with SGAs and antidepressants being most commonly dispensed.

**Conclusion:**

Despite lithium's efficacy and status as first‐line BD maintenance treatment, its dispensing in Dutch youth remains low compared to the BD prevalence. This may be due to misdiagnosis of BD and/or clinicians' reluctance to prescribe lithium, highlighting the need for improved diagnostics, clinician education, and further research into lithium's safety in youths.

## 1 Introduction

Bipolar disorder is a complex and severe psychiatric mood disorder, characterized by recurrent (hypo)manic and depressive episodes, with a worldwide prevalence of 2.4% [1]. It is associated with a significant decrease in overall quality of life, functional disability, social stigma, and a high risk of recurrence [2, 3, 4]. Although bipolar disorder can manifest across the lifespan, the peak age of onset typically occurs during mid‐adolescence and early adulthood [5, 6]. Early‐onset bipolar disorder is a key contributer to educational dysfunction and increases the risk of comordib conditions, including substance use, anxiety disorders, suicidical behavior, and an increased frequency of depressive and manic episodes. Additionally, it is linked to a chronic illness trajectory that often persists into adulthood [7, 8].

Despite increasing recognition of bipolar disorder occurence in youth, there remains a lack of research examining the global burden of bipolar disorder in younger individuals. Epidemiological data on the prevalence of bipolar disorder in youth remain limited in the Netherlands. A recent study by Zhong et al. reported a prevalance of 865 per 100,000 youths (0.87%) among individuals aged 10 to 24 years in the Netherlands in 2019 [9]. Notably, pediatric bipolar disorder (under 12 years) is rarely diagnosed in the Netherlands, largely due to sceptism regarding the diagnostic validity in prepubertal children [10, 11]. Globally, there is substantial evidence indicating that bipolar disorder is frequently undertreated. In the United States, a study by Merikangas et al. found that approximately 2.2% of adolescents have a bipolar spectrum disorder, yet only 20%–50% of these individuals receive any form of treatment [12]. Similarly, a French study revealed that patients with early‐onset bipolar disorder (before age 21) experienced an average duration of untreated illness of 12.5 years [13]. Delay in accurately diagnosing bipolar disorder and initiating appropriate treatment can lead to significant consequences, including poor social adjustment, disruption of critical developmental tasks, employment challenges, suboptimal response to mood‐stabilizing treatments, more frequent rapid cycling, higher hospitalization rates, and an elevated risk of suicide. These outcomes highlight the importance of early recognition and adequate treatment of bipolar disorder [14].

Lithium, a well‐established mood‐stabilizer with antimanic, antidepressant, and anti‐suicidal properties, remains a cornerstone and first‐line treatment in the maintenance phase of bipolar disorder [15, 16, 17]. In youth, evidence suggests that lithium therapy is associated with superior long‐term outcomes, reduced duration of depressive episodes, and lower suicide rates compared to SGAs, and antimanic anticonvulsants [18]. Furthermore, earlier initiation of lithium treatment has been linked to improved treatment response [19]. Despite its proven efficacy, recent epidemiological studies report a decline in lithium use among children, adolescents and adults, while the use of SGAs in bipolar patients is rising over time [20, 21, 22]. This trend may stem from limited clinical experience with lithium, partly due to the low prevalence of bipolar disorder. Furthermore, both clinicians and patients may overestimate lithium's potential long‐term side effects, such as cognitive impairment, kidney or thyroid dysfunction. Moreover, its narrow therapeutic window, requiring regular blood monitoring and posing a risk of toxicity, could present an extra burden for clinicians and patients [23]. In contrast, the substantial risks of SGAs for age‐inappropriate weight gain, cardiometabolic side effects, and hyperprolactinemia are often underestimated. Especially cardiometabolic side effects in children and adolescents present a serious concern, as youths are highly at risk for the metabolic changes triggered by SGAs. Such changes can lead to long‐term health consequences, including adult obesity, diabetes type 2, metabolic syndrome, and cardiovascular morbidity [24].

Considering the potential underutilization of lithium treatment in younger populations, pharmaco‐epidemiological research is crucial to assess the patterns of lithium dispensing, along with concomitant and preceding medication use, to better understand treatment trends in youth. To the best of our knowledge, no other studies have reported the prevalence or incidence of lithium usage in pediatric and young adult populations in the Netherlands. Therefore, this study aims to investigate trends in lithium dispensing among children, adolescents, and young adults in the Netherlands between 2011 and 2022. The analysis will evaluate prevalence, incidence, dosages, duration of use, and patterns of concomitant and prior psychotropic medication.

## 2 Method

### 2.1 Data Source

The study was performed with pharmacy dispensing data collected from the population‐based prescription database IADB.nl of University of Groningen [25]. The IADB.nl database collects prescription drug dispensing data from community pharmacies in the northern and eastern part of the Netherlands, starting from 1994, through an opt‐out approach. These pharmacies cover data of approximately 1,300,000 patients, which are representative for the general Dutch population [26]. Approval of the medical ethics committee was not required since the data and records in the IADB.nl database is anonymized. Additionally, data collection complies with both national and European privacy standards for handling human data.

### 2.2 Study Sample

Patients aged 6 through 25 years who were dispensed lithium at least once between January 1, 2011, and December 31, 2022, were selected. The dataset contained general information, including year of birth, sex, dates of inclusion in the database, and yearly population estimates, as well as detailed dispensing information, such as the date of dispensing, quantity supplied, daily dose, and defined daily dose (DDD). Lithium was categorized as N05AN based on the World Health Organization's Anatomical Therapeutic Chemical/Defined Daily Dose (ATC/DDD) Classification System. In children up to 18 years, lithium is exclusively registered and indicated for the treatment of bipolar disorder, whereas in adults, it is also indicated as an augmentation therapy for unipolar depression in combination with selective serotonin reuptake inhibitor (SSRI) or tricyclic antidepressant (TCA) [17, 27]. To assess the indication for lithium dispensing and co‐medication patterns, data were retrieved based on ATC codes for antidepressants (N06A), antipsychotics (N05A, excluding lithium), anxiolytics (N05B), hypnotics and sedatives (N05C), psychostimulants (N06B), psycholeptics and psychoanaleptics in combination (N06C), valproic acid (N03AG01), and lamotrigine (N03AX09) [28]. These medications were selected for their relevance in the treatment of psychiatric disorders commonly managed alongside lithium therapy [17], providing insights into comorbidities, potential drug interactions, and dispensing patterns.

### 2.3 Data Analysis

#### 2.3.1 Prevalence and Incidence

Prevalence and incidence rates of lithium use were calculated per year from 2011 to 2022. The ages in the database were based on the first of January of the year of the dispensing. Incident users were defined as individuals who initiated lithium dispensing during the study period and had no record of corresponding dispensations in the 365 days preceding the index date. The index date was defined as the date of the first lithium dispensing within the study period. To calculate prevalence and incidence rates, the number of users was divided by the total underlying population present in the IADB.nl database and presented per hundred thousand individuals. The total underlying population is an annual estimate and can be viewed as a dynamic stationary cohort without substantial changes in size throughout the year, ranging from 276,834 to 300,619 individuals aged 6–25 years between 2011 and 2022. The rates were further stratified by sex and age groups (6–12 years, 13–19 years, and 20–25 years), as these variables are critical for capturing demographic differences. The chosen age categories align with distinct developmental, educational, and social stages, corresponding to middle child, adolescence, and early adulthood, respectively.

#### 2.3.2 Dose Analysis

Dose analysis was performed, where median daily dosages per year were analyzed using defined daily dose (DDD) [28]. This analysis was restricted to individuals prescribed lithium carbonate. The DDD used for calculations was 24 mmol lithium, equivalent to 888.89 mg lithium carbonate. The age on the first of January of the year of dispensation was used. The median daily doses are presented as the value with the interquartile range (IQR). Only dispensations issued for at least 7 days were selected to exclude rescue medication.

#### 2.3.3 Duration of Lithium Use

The duration of lithium use for incidence users was calculated in months based on the median survival times estimated using the Kaplan Meier estimator. The start of a lithium use period was defined as the initial dispensation. Lithium treatment was considered discontinued if the total duration of lithium dispensation, plus an additional 90 days, had elapsed without subsequent dispensation, while the patient remained in the database. This approach accounted for potential temporary nonadherence or an acute manic episode necessitating hospitalization and/or SGA treatment. All other individuals were classified as censored. When an individual turned 26 during the study period, they were automatically excluded from the analysis. The median survival times were calculated for sex and age groups (at time of start of lithium dispensing). Starters from the year 2022 were excluded from the survival analysis to avoid censoring bias, as the cohort could only be followed until the end of 2022, and in this year high rates of censoring would take place. Duration of lithium use was presented in months, with 30 days being considered 1 month.

#### 2.3.4 Preceding and Concurrent Psychotropic Medication

An analysis of last prior and concurrent psychotropic medication was performed to understand the potential indication for lithium, associated comorbidities, adherence to guidelines, and treatment decision‐making patterns. Last prior medication included dispensations before the first lithium prescription, while concurrent treatment was defined as psychotropic dispensing overlapping with lithium use for at least 90 days, with the median duration of overlap calculated. Co‐medications were analyzed by distinguishing between cases where lithium was combined with a single psychotropic medication and those involving multiple co‐medications. Similarly, last prior psychotropic use prior to lithium was categorized into single and multiple medications, requiring a minimum use of 7 days for inclusion. Additionally, we calculated the median duration of the last prior prescription until the initiation of lithium.

#### 2.3.5 Statistical Analysis

All analyses were performed using R, version 4.3.1, with a two‐tailed significance threshold of 0.05 for all statistical tests. The mean, median and 95% confidence interval of each variable were also estimated. The Kruskal‐Wallis test was used to compare group medians, and the *t*‐test was used for means comparisons. For categorical variables, the chi‐squared test was applied.

## 3 Results

A total of 612 individuals aged 6–25 years were dispensed lithium at least once anywhere during the period 2011 to 2022. The cohort was stratified into 13 individuals aged 6–12 years, 90 individuals aged 13–19 years, and 509 individuals aged 20–25 years, with 262 males and 350 females.

### 3.1 Prevalence

**FIGURE 1:**
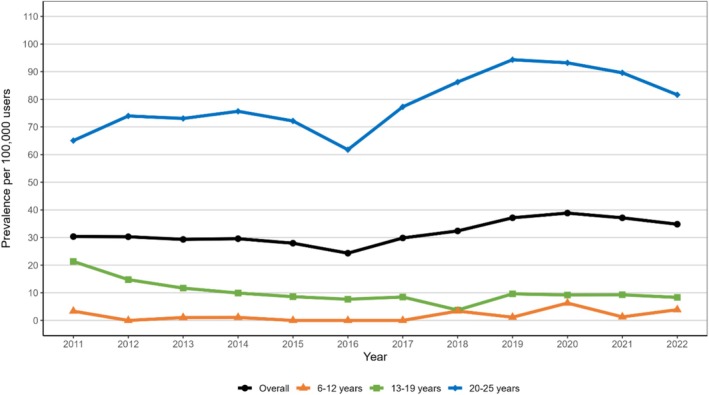
Overall and age group‐specific prevalence of lithium dispensations per 100,000 in individuals aged 6 to 25 years between 2011 and 2022.

The yearly total prevalence rate of lithium dispensing among youth changed over time, from 30.3 per 100,000 in 2011 to 34.8 per 100,000 in 2022, with no significant overall linear trend between 2011 and 2022 (*p* = 0.40). A decline in lithium use among youth was observed between 2011 and 2016, from 30.3 per 100,000 in 2011 to 24.3 per 100,000 in 2016 (*p* = 0.19). Following 2016, a significant increase in prevalence was observed, reaching its peak in 2020 at 38.8 per 100,000 (*p* < 0.001) (Figure 1). However, since 2020, a slight decrease in prevalence has been noted, reaching a total prevalence of 34.8 per 100,000 in 2022 (*p* = 0.48). The prevalence rates stratified by age, sex and year are presented in Table 1.

**TABLE 1:** Prevalence (per 100,000) of lithium dispensations among children, adolescents, and young adults between 6 and 25 years in 2011 and 2022.

Total	2011^a^ per 100,000	2022^b^ per 100,000	*p*
All age groups	30.3	34.8	0.40
6–12 years	3.4	3.9	1.00
13–19 years	21.3	8.3	0.03 ↓
20–25 years	65.1	81.6	0.20
*Males*			
All age groups	23.5	29.9	0.36
6–12 years	4.4	5.1	1.00
13–19 years	19.4	4.1	0.03 ↓
20–25 years	47.7	72.6	0.16
*Females*			
All age groups	36.9	39.8	0.77
6–12 years	2.3	2.6	1.00
13–19 years	23.2	12.7	0.33
20–25 years	80.4	90.7	0.65

*Note:* ↓ = significant decrease.

^a^ *n* = 136,081 males, *n* = 140,753 females, *n* = 276,834 total lithium users.

^b^ *n* = 139,165 males, *n* = 140,967 females, *n* = 280,132 total lithium users.

**FIGURE 2:**
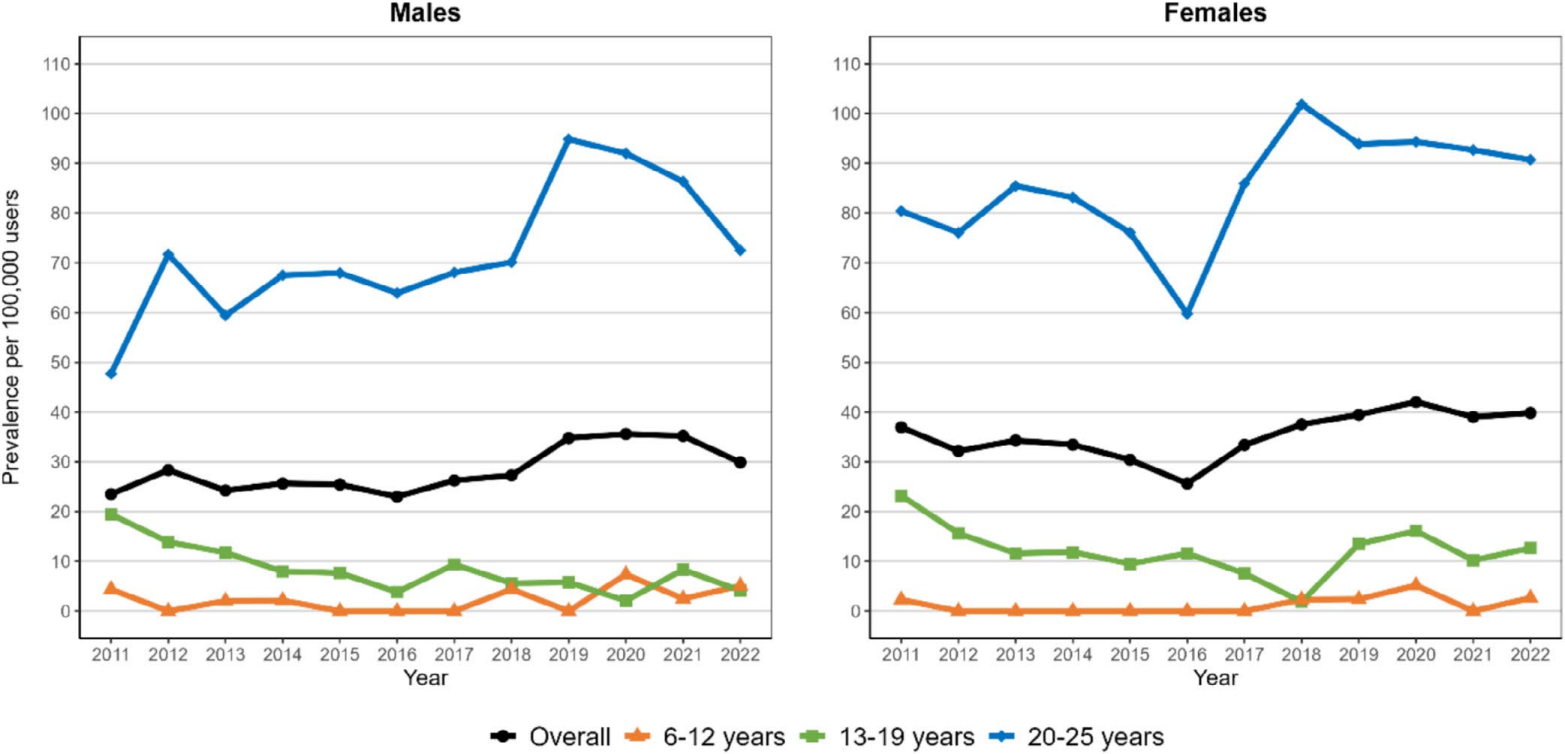
Overall and age group‐specific prevalence of lithium dispensations per 100,000 users among males and females.

Regarding age and sex, the lowest prevalence rate was observed in the 6–12 age category, reaching a peak of 6.3 users per 100,000 in 2020. In the other years, prevalence within this age category ranged from 0 to 3.9 per 100,000 users, with no significant difference between 2011 and 2022 (*p* = 1.00). Notably, the youngest individuals dispensed lithium were as young as 6 years old (*n* = 1). Furthermore, no significant differences were observed between males and females in this age group. Among adolescents aged 13–19, prevalence rates peaked at 21.3 per 100,000 users in 2011, then declined to a low of 3.8 per 100,000 in 2018, before rising again to 9.6 per 100,000 in 2019, after which it remained steady. No significant differences between males and females were noted in the 13–19 age group overall. However, from 2019 onward, prevalence increased significantly in females compared to males (*p* = 0.03). Among young adults aged 20–25, the highest prevalence rates across all age groups were observed, increasing from 65.1 to 81.6 per 100,000 between 2011 and 2022 (*p* = 0.20). The lowest rate of 61.7 was observed in 2016, while the highest rate of 94.3 was observed in 2019. Females had significantly higher prevalence rates compared to males in the 20–25 age group (*p* = 0.02). Across all age groups combined, the prevalence rate was significantly higher in females than in males (*p* = 0.03). Figure 2 displays the prevalence of lithium dispensations by age group and overall, stratified by sex.

### 3.2 Incidence

**FIGURE 3:**
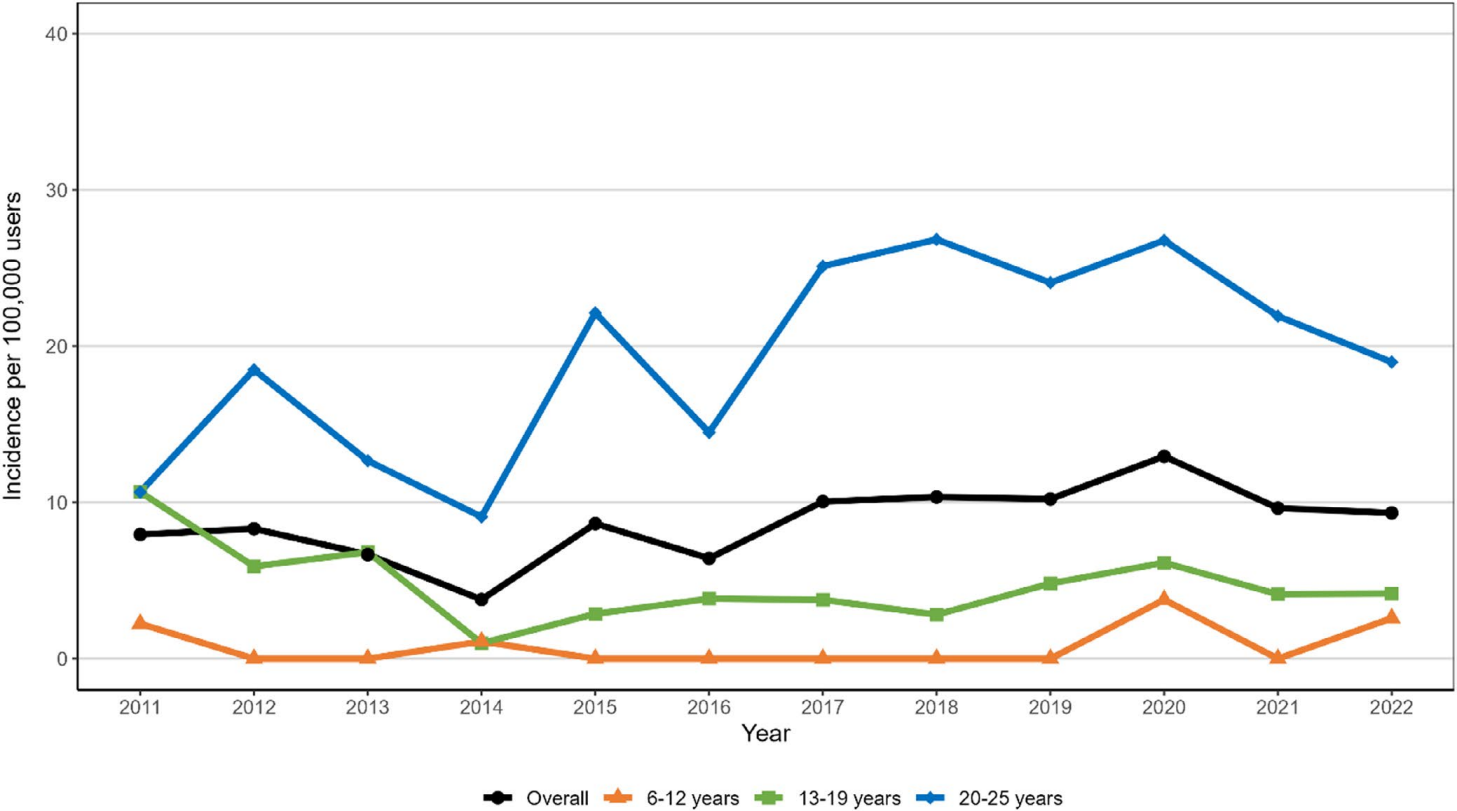
Overall and age‐group specific incidence of lithium dispensations per 100,000 in individuals aged 6–25 years between 2011 and 2022.

**FIGURE 4:**
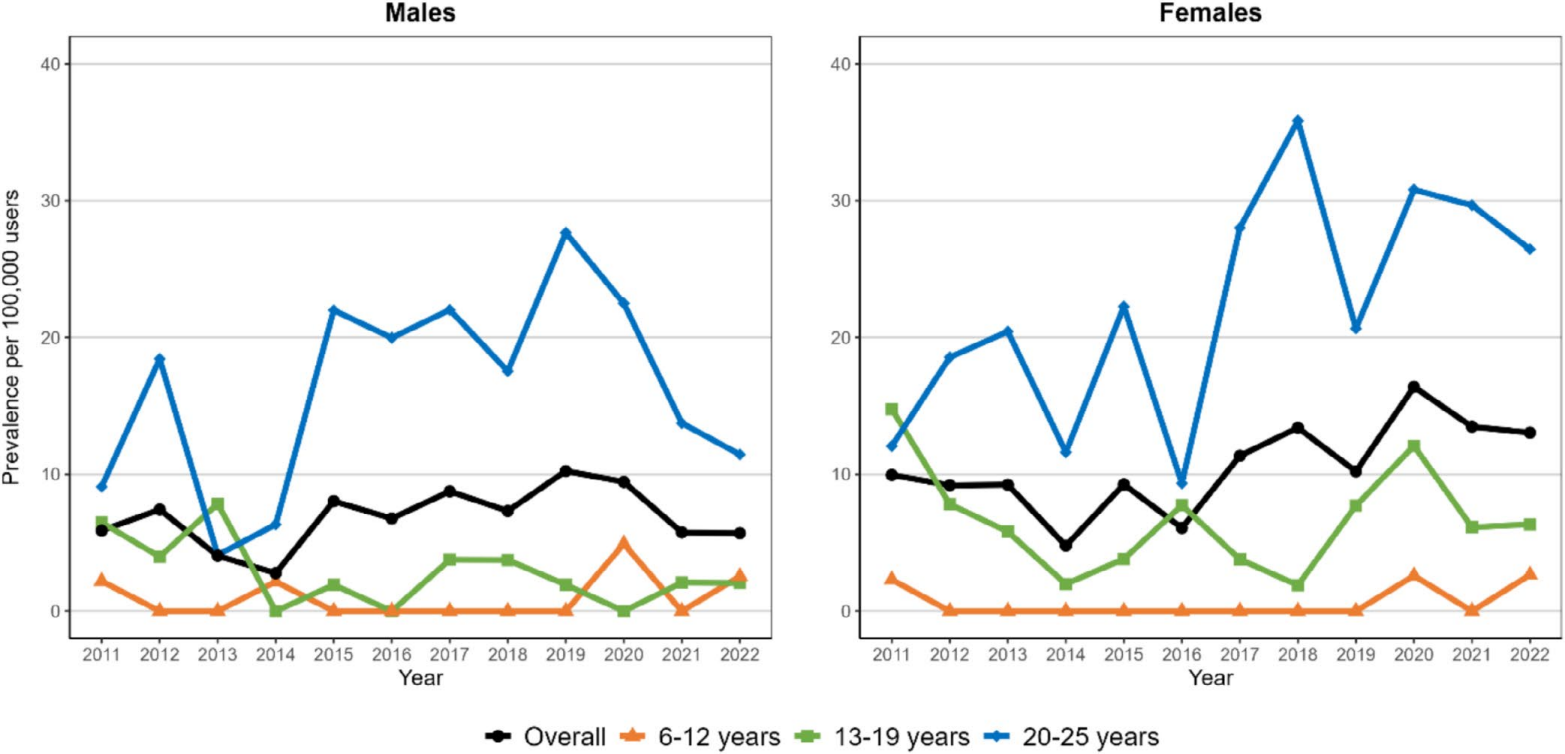
Overall and age‐specific incidence of lithium dispensations per 100,000 users among males and females.

The overall incidence was 7.9 per 100,000 in 2011 and 9.3 per 100,000 in 2022 (*p* = 0.68), with a peak of 12.9 per 100,000 in 2020 among users aged 6 to 25 years, as shown in Figure 3. For the 6–12 age group, new users were recorded only in 2011, 2014, 2020 and 2022, with rates ranging between 1.1 and 3.8 per 100,000. In 2011, the incidence rate for both the 13–19 and 20–25 age groups was 10.7 per 100,000. However, for the 13–19 age group, this rate declined over the following years, reaching a significant low of 1.0 per 100,000 in 2014, before gradually increasing to 4.2 in 2022. In contrast, the 20–25 age group experienced a continuous increase in incidence rates following 2011, peaking at 26.8 per 100,000 in 2018 and subsequently reaching 19.0 per 100,000 in 2022. Overall, females had a higher incidence rate compared to males (*p* = 0.006), particularly in the 13–19 (*p* = 0.02) group (Figure 4). The incidence rates, stratified by age, sex, and start year are presented in Table 2.

**TABLE 2:** Incidence (per 100,000 individuals) of lithium dispensations among children, adolescents, and young adults between 6 and 25 years in 2011 and 2022.

Total	2011^a^ per 100,000	2022^b^ per 100,000	*p*
All age groups	8.0	9.3	0.68
6–12 years	2.2	2.6	1.00
13–19 years	10.7	4.2	0.11
20–25 years	10.7	19.0	0.18
*Males*			
All age groups	5.9	5.8	1.00
6–12 years	2.2	2.5	1.00
13–19 years	6.5	2.1	0.36
20–25 years	9.1	11.5	0.76
*Females*			
All age groups	10.0	13.0	0.56
6–12 years	2.2	2.6	1.00
13–19 years	14.8	6.3	0.34
20–25 years	12.0	26.5	0.15

^a^ *n* = 136,081 males, *n* = 140,753 females, *n* = 276,834 total people.

^b^ *n* = 139,165 males, *n* = 140,967 females, *n* = 280,132 total people.

### 3.3 Dosage Analysis

A total of 1069 lithium carbonate dispensations were recorded in the database. From these data, the median dosage of lithium (in milligrams) was calculated and stratified by year and age group. The median dosage was 922 mg (IQR: 785–1114 mg) in 2011, and gradually declined to 776 mg (IQR: 555–1000 mg) by 2022. Dosage varied significantly across age category: children aged 6–12 years received the lowest median dosage (215 mg), adolescents aged 13–19 years and young adults aged 20–25 years both received median dosage of 800 mg. Males received significantly higher median dosages of lithium carbonate (925 mg) compared to females (797 mg, *p* < 0.001).

### 3.4 Duration of Use

**FIGURE 5:**
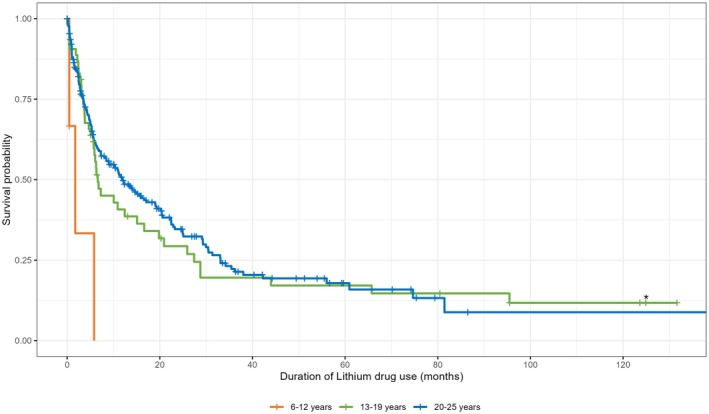
Duration of lithium use per age group. *Each vertical line indicates a person that has stopped using lithium.

**FIGURE 6:**
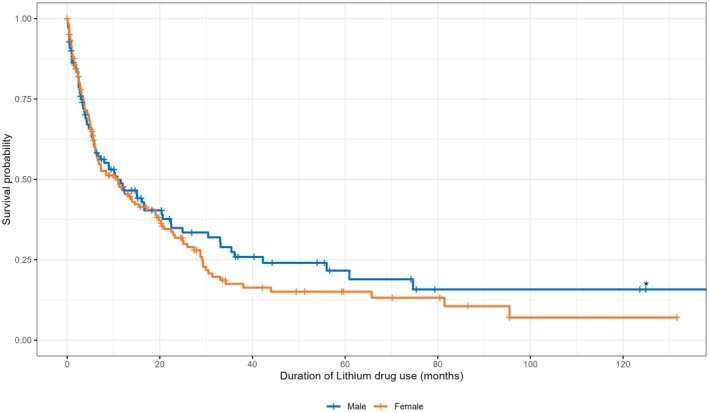
Duration of lithium use for males and females. *Each vertical line indicates a person that has stopped using lithium.

The duration of lithium use was analyzed by both age category and sex, as shown in Figures 5 and 6. Among the age groups, children aged 6–12 years had the shortest median duration of use at 0.5 months (*n* = 6). In comparison, adolescents aged 13–19 years (*n* = 54), and young adults aged 20–25 years (*n* = 217) had significantly longer median durations of 6.2 months and 8.6 months, respectively. Notably, all the users aged 6–12 years discontinued lithium within 10 months, whereas the older age groups continued its use for up to approximately 12.5 years. Regarding sex, males had a longer median duration of use at 7.5 months compared to 6.6 months for females; however, this difference was not statistically significant (*p* = 0.79).

### 3.5 Concurrent and Preceding Psychotropic Medication

More than half of the cohort (55%) had been dispensed other psychotropic medications prior to initiating lithium treatment, while 52% received other psychotropic medications concurrently with lithium treatment. The most commonly dispensed psychotropic drug class, both concurrently and prior to lithium therapy, were SGAs, particularly quetiapine and olanzapine. In addition to SGAs, methylphenidate, and SSRIs, such as fluoxetine and citalopram, were most often dispensed before lithium dispensation. Tables 3 and 4 provide an overview of the most commonly dispensed psychotropic drugs prior to and concurrent with lithium dispensation, respectively. The proportion of females using comedication (61%) was comparable to that of males (56%), with no statistically significant difference observed (*p* = 0.29). Additionally, we found that 31% of the last SGA dispensations exceeded 12 weeks before the initiation of lithium.

**TABLE 3:** The most commonly dispensed psychotropic medication immediately prior to lithium dispensing, by age group, and distinguishing between monotherapy and polypharmacy, in the Dutch population between 2011 and 2022.

Age group	Top 3 last prior psychotropic medications (n, median duration last prescription until lithium use)	
**6–12 years (*n* = 13)**	**Monotherapy (*n* = 10)**	**Combination therapy (*n* = 0)**
	Quetiapine^AP^ (1, 70 days) Venlafaxine^AD^ (1, 28 days) Citalopram^AD^ (1, 22 days)	N/A
**13–19 years (*n* = 90)**	**Monotherapy (*n* = 67)**	**Combination therapy (*n* = 6)**
	Olanzapine^AP^ (15, 42 days) Quetiapine^AP^ (8, 44 days) Fluoxetine^AD^ (5, 108 days)	Quetiapine^AP^—Aripiprazole^AP^ (1, 165 days) Quetiapine^AP^—Risperidone^AP^—Clomipramine^AD^ (1, 143 days) Valproic acid^AE^—Aripiprazole^AP^ (1, 64 days)
**20–25 years (*n* = 509)**	**Monotherapy (*n* = 225)**	**Combination therapy (*n* = 27)**
	Olanzapine^AP^ (48, 32 days) Quetiapine^AP^ (47, 33 days) Aripiprazole^AP^ (14, 179 days)	Quetiapine^AP^—Escitalopram (2, 188 days) Venlafaxine^AD^—Aripiprazole^AP^ (1, 284 days) Quetiapine^AP^—Aripiprazole^AP^—Clomipramine^AD^ (1, 270 days)

*Note:* The table presents the top three medications for both monotherapy and polypharmacy, including the number of users (*n*) and the median duration of the last prescription before lithium dispensing.

Abbreviations: AD, antidepressants; AE, antiepileptic; AP, antipsychotics; *n* = individuals; N/A, not applicable; PS, psychostimulant.

**TABLE 4:** Most commonly dispensed psychotropic medications concurrent with lithium use in the Dutch population (2011–2022), stratified by age group and treatment regimen.

Age group	Top 3 concurrent psychotropic medications (n, median overlapping days with lithium)	
**6–12 years** **(*n* = 13)**	**One concurrent medication (*n* = 0)**	**Multiple concurrent medications (*n* = 0)**
**13–19 years** **(*n* = 90)**	**One concurrent medication (*n* = 28)**	**Multiple concurrent medications (*n* = 13)**
	Olanzapine^AP^ (7, 124 days) Quetiapine^AP^ (5, 186 days) Aripiprazole^AP^ (4, 411 days)	Aripiprazole^AP^—Quetiapine^AP^ (2, 260 days) Citalopram^AD^—Haloperidol^AP^—Paliperidone^AP^ (1, 406 days) Clozapine^AP^—Valproic acid^AE^ (1, 403 days)
**20–25 years (*n* = 509)**	**One concurrent medication (*n* = 184)**	**Multiple concurrent medications (*n* = 95)**
	Olanzapine^AP^ (46, 159 days) Quetiapine^AP^ (32, 229 days) Aripiprazole^AP^ (15, 281 days)	Lamotrigine^AE^—Quetiapine^AP^ (5, 141 days) Sertraline^AE^—Quetiapine^AP^ (4, 271 days) Haloperidol^AP^—Quetiapine^AP^ (4, 257 days)

*Note:* The table presents the three most commonly prescribed psychotropic medications used alongside lithium, categorized by single versus multiple concurrent psychotropic medications. For each medication or combination, the number of users (*n*) and the median duration of concurrent use (measured in overlapping days) are reported.

Abbreviations: AD, antidepressants; AE, antiepileptic; AP, antipsychotics; *n*, individuals.

**FIGURE 7:**
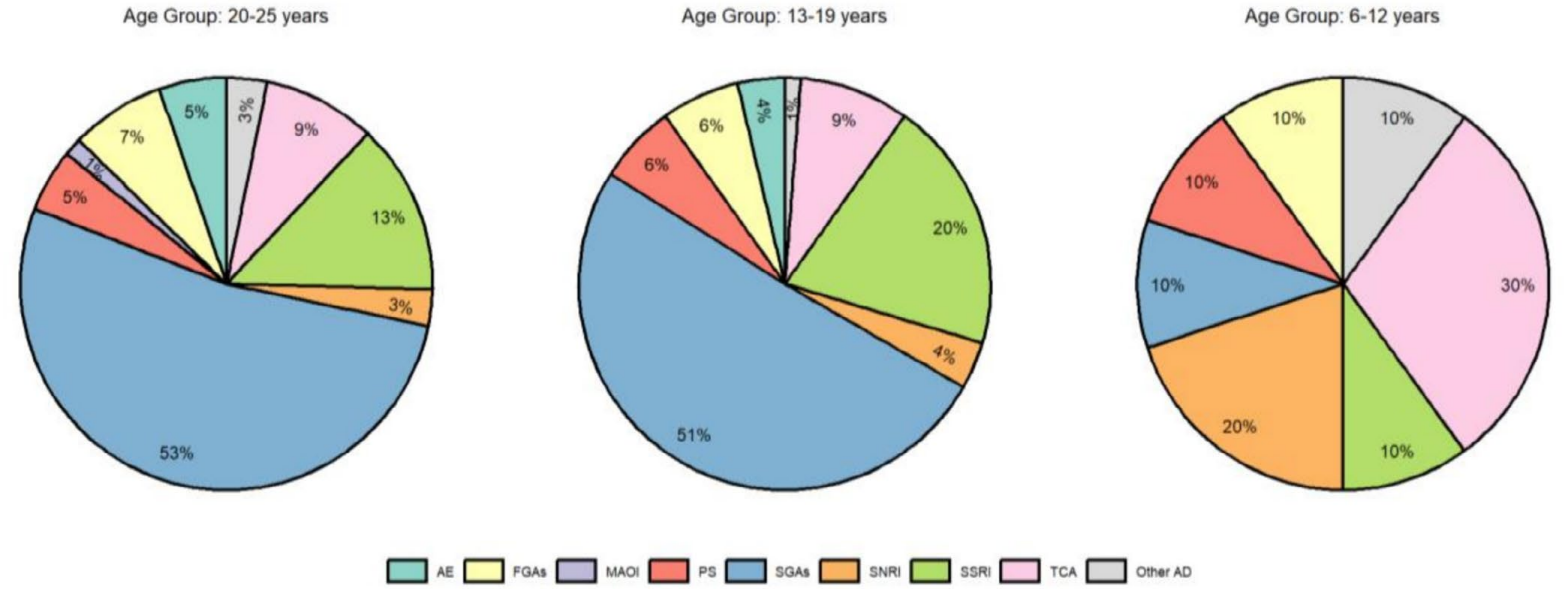
Distribution of the last psychotropic medication dispensed prior to lithium initiation, stratified by age group, in the Dutch population (2011–2022). AE, antiepileptics; FGAs, first‐generation antipsychotics; MAOI, monoamine oxidase inhibitors; PS, psychostimulants; SGAs, second‐generation antipsychotics; SNRI, serotonin‐norepinephrine reuptake inhibitors; SSRI, selective serotonin reuptake inhibitors; TCA, tricyclic antidepressants; Other AD, other antidepressants.

The pie charts in Figure 7 show the relative proportions of different classes of psychotropic medications prescribed immediately before lithium initiation across three age groups (6–12 years, 13–19 years, and 20–25 years). SGAs were the most commonly prescribed in the 13–19 and 20–25 age groups, while tricyclic antidepressants (TCAs) were most common in the 6–12 age group.

**FIGURE 8:**
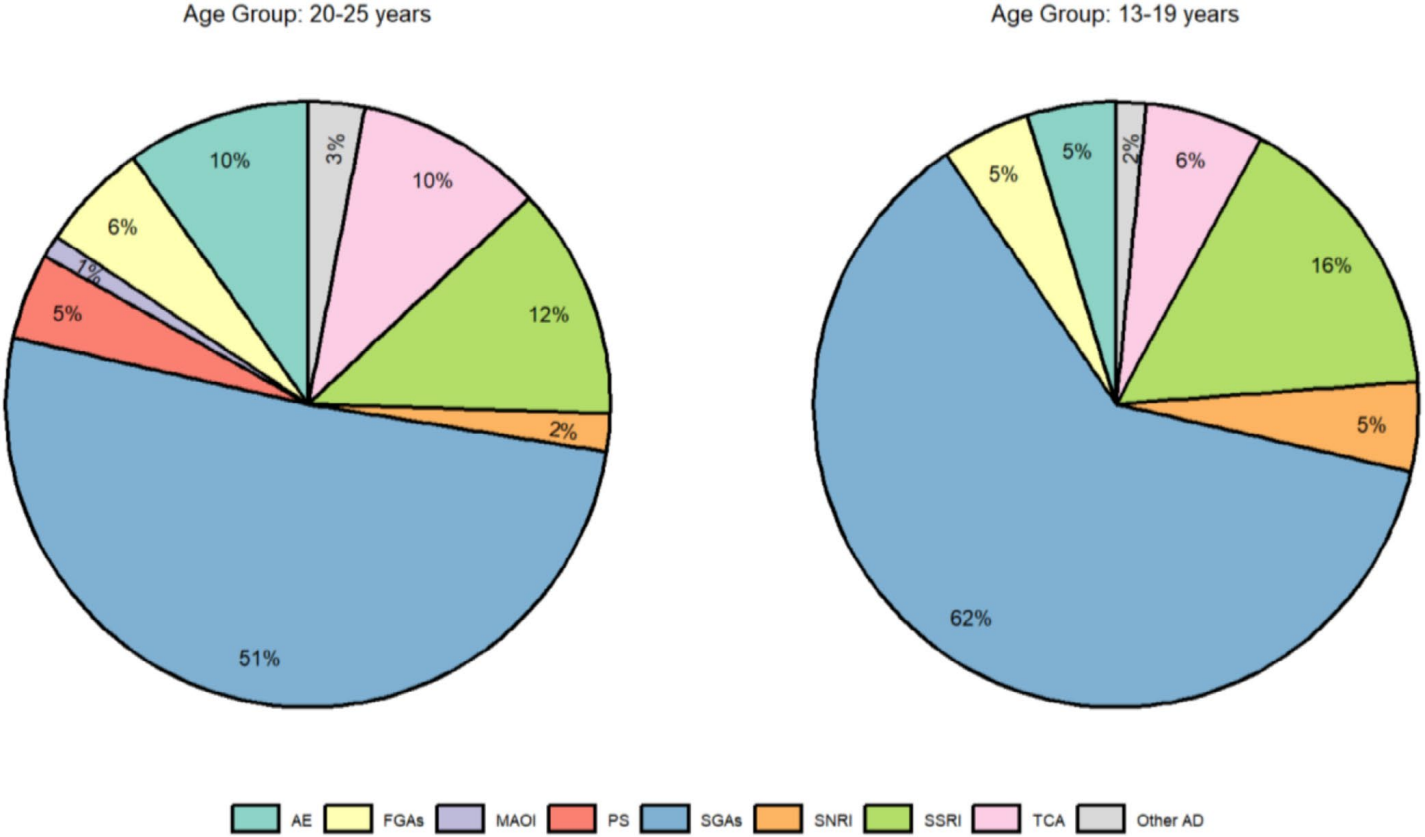
Distribution of the most commonly dispensed psychotropic medications concurrent with lithium use, stratified by age group, in the Dutch population (2011–2022). AE, antiepileptics; FGAs, first‐generation antipsychotics; MAOI, monoamine oxidase inhibitors; PS, psychostimulants; SGAs, second‐generation antipsychotics; SNRI, serotonin‐norepinephrine reuptake inhibitors; SSRI, selective serotonin reuptake inhibitors; TCA, tricyclic antidepressants; Other AD, other antidepressants.

Figure 8 illustrates the distribution of psychotropic medication classes prescribed alongside lithium for individuals aged 13–19 years and 20–25 years. Notably, no psychotropic medication was prescribed alongside lithium for at least 90 days in the 6–12 age group. The most commonly prescribed medications concurrent with lithium were SGAs.

## 4 Discussion

From 2011 to 2022, the yearly prevalence of lithium dispensing among 6–25‐year‐olds in the Netherlands fluctuated, ranging from 30.3 per 100,000 in 2011 to 34.8 per 100,000 in 2022, with a low of 24.3 per 100,000 in 2016 and a peak of 38.8 per 100,000 in 2020. The highest prevalence of lithium dispensing was observed among young adults aged 20–25 years, increasing from 65.1 per 100,000 in 2011 to 81.7 per 100,000 in 2022 (*p* = 0.20). In contrast, a significantly lower prevalence was observed among adolescents aged 13–19 years (*p* < 0.001), with prevalence rates significantly dropping from 21.3 per 100,000 in 2011 to 8.3 per 100,000 in 2022 (*p* = 0.03).

The prevalence of lithium dispensing relative to the prevalence of bipolar disorder is notably lower. A study by Zhong et al. estimated the prevalence of bipolar disorder in children aged 10 to 24 years at 0.87% in 2019. In contrast, our findings show a much lower prevalence rate of 0.037% for lithium dispensing within the same population during that year. This suggests a potential underutilization of lithium as a treatment for bipolar disorder in youths in the Netherlands. We contend that this may be due to either underdiagnosis of bipolar disorder or a reluctance to initiate lithium treatment.

Bolton et al. reported that the majority of individuals with bipolar disorder emerge in early‐life, typically between ages 14 and 21, with a mean age of onset around 17 years [6]. In contrast, the higher peak age of initial lithium dispensation observed in our study suggests a diagnostic in adolescent. This is consistent with previous findings, which suggest that individuals with early‐onset bipolar disorder (before age 21) often endure prolonged periods of untreated illness, with an average untreated duration up to 16 years [13, 29]. A major contributor to this delay is the overlap of symptoms between early bipolar disorder and other psychiatric conditions [30, 31]. Studies have shown that manic symptoms, depression and emotion regulation difficulties in pediatric bipolar disorder (6–12 years) often overlap significantly with the presentation of Attention Deficit Hyperactivity Disorder (ADHD) [10]. This can lead to an initial misdiagnosis and the prescription of ADHD medications. Our findings further align with this pattern, as we observed that some patients in the 6–12 and 13–19 age groups received methylphenidate monotherapy before switching to lithium. Additionally, we found frequent dispensation of antidepressants prior to lithium monotherapy in the 13–19 and 20–25 age groups, often in combination with SGAs. Since depression is often the initial presentation in bipolar disorder in individuals with an onset before age 25, delayed differentiation from unipolar depression is common [32, 33]. This dispensing pattern aligns with clinical guidelines, which recommend SSRIs for acute bipolar depression when combined with lithium or olanzapine [17, 34], as well as augmentation therapy in which lithium is added to either an SSRI or TCA for unipolar depression in adults [27]. However, antidepressant treatment can trigger manic episodes in individuals with unrecognized bipolar disorder, particularly when risk factors such as family history of bipolar disorder [35], recurrent episodes of depression during youth, psychotic or hypomanic features accompanying depressive episodes [36], and treatment‐resistant depression [37] are present. Diagnostic uncertainty in younger populations, particularly the frequent overdiagnosis of ADHD and major depressive disorder and the underdiagnosis of bipolar disorder, may contribute to clinicians' reluctance to prescribe lithium, ultimately delaying appropriate treatment initiation [38].

Safety concerns surrounding lithium use in younger populations, along with the requirement for frequent blood monitoring, may also lead to underutilization of lithium. Clinicians may be reluctant to prescribe lithium and instead opt for alternative medications, such as SGAs [39, 40]. This possible reluctance is further supported by our findings, which reveal a prevailing pattern of SGA dispensing prior to lithium dispensing, particularly among the age groups 13–19 and 20–25 years. Quetiapine, olanzapine, and aripiprazole were the most commonly dispensed psychotropic drugs in our study population, consistent with both Dutch and National Institute for Health and Care Excellence (NICE) guidelines that recommend SGAs as first‐line treatment for acute (hypo)manic episodes, especially in patients not yet on maintenance therapy [17, 34]. However, these guidelines also advice limiting antipsychotic treatment to 12 weeks in young people, with gradual tapering during the maintenance phase, since long‐term use of SGAs is associated with metabolic side effects [17, 34]. Despite these recommendations, our findings show that approximately 32% of patients who received SGAs before their first lithium dispensation used them for over 12 weeks. Additionally, nearly half of the patients were concurrently dispensed an SGA alongside lithium. While the concomitant use of lithium and SGAs has been shown to effectively manage acute manic symptoms, the prolonged use of this combination raises concerns as this could reflect the severity of early onset bipolar disorder and the typically more psychotic/mixed symptoms which could require longer use of SGAs. We observed that the simultaneous use of lithium and SGAs lasted for extended periods, ranging from a median of 17 to 59 weeks, depending on the specific antipsychotic drug. Another possible explanation for the selection of SGAs over lithium, despite lithium being the first‐line maintenance therapy for bipolar disorder, may stem from prescribing practices influenced by diagnostic and therapeutic considerations. Clinicians may initially misinterpret symptoms as psychosis rather than bipolar disorder, especially in early‐onset cases where manic episodes often present with psychotic symptoms, leading to the selection of antipsychotics [41]. These factors further highlight the need for greater emphasis on accurate diagnosis, adherence to tapering protocols, and a more rapid transition to the maintenance phase when necessary.

Regarding duration of use, the median lithium dispensation period in the small cohort of patients aged 6–12 years was remarkably short, with a median use of only 0.5 months and a maximum observed duration of 7 months. This short duration of use, combined with the low prevalence rates of lithium dispensing, and the wide range of preceding psychotropic medications and co‐medications in this age group highlight the frequent misdiagnosis of bipolar disorder and the significant challenges in accurately diagnosing psychiatric disorders in pediatric populations. In contrast, the duration of use in the 13–19 and 20–25 age group was 6.2 and 8.6 months, respectively. Notably, 22% of patients in these groups continued lithium treatment for at least 2 years, with some individuals receiving lithium for as long as 10 years. The variability of duration of use may be explained by the differing profiles of lithium responders and non‐responders. Previous research indicates that lithium responders typically have stable long‐term treatment responses, a recurrent episodic illness, and fewer comorbidities. Additionally, their affected relatives often demonstrate positive responses to lithium treatment [42]. It is essential for clinicians to consider these factors when determining lithium treatment, ensuring that patients who may be potential lithium responders are given the opportunity to receive it.

Additionally, we observed a significant difference in the prevalence and incidence of lithium dispensations between males and females, with females showing higher rates, particularly in the 13–19 and 20–25 age groups after 2019. This finding is consistent with a study conducted in France, which reported that females aged 6–25 received more lithium prescriptions than males from 2020 to 2023, continuing a trend that was already evident before 2020 [43]. However, other studies have reported similar prevalence rates of bipolar disorder among males and females, with no substantial sex‐based distinctions [44]. The higher dispensing rates in females may reflect sex differences in symptom presentation, help‐seeking behavior, healthcare utilization, or prescribing practices during adolescence and young adulthood, rather than differences in bipolar disorder prevalence itself. Furthermore, it is noteworthy that males were dispensed significantly higher mean doses of lithium carbonate compared to females (*p* < 0.001). This is likely due to differences in body weight, renal function, or other factors that influence lithium's pharmacokinetics, as lithium requires dosage adjustments within its narrow therapeutic window. However, this could not be identified with the available data.

Possible factors that also may have influenced the prevalence and incidence of lithium dispensations, were the introduction of the Youth Act in 2015 in the Netherlands and the coronavirus disease 2019 (COVID‐19). The Youth Act, which shifted the responsibility for youth care from the national government to local municipalities with a focus on ‘de‐medicalization’, early intervention, and prevention, may have influenced the approach to diagnosing and treating mental health conditions, potentially affecting the prescription of psychotropic drugs. Previous research on the impact of the Youth Act on psychotropic dispensing patterns has demonstrated a reduction in ADHD medication dispensations in youths following its implementation [45]. However, no statistically significant changes were observed in the dispensation rates of antipsychotics [46] among individuals aged 0 to 19. The extent to which the Youth Act has influenced lithium dispensations in our study, however, remains unclear, as the law primarily targets individuals up to 18 years old, a group that represents a smaller portion of the population in our study. The slight decline in prevalent and incidence numbers after 2019 must be considered in the context of COVID‐19 pandemic, as the pandemic and subsequent policy responses affected access to healthcare services. Unobstructed access to health care is crucial for lithium treatment, due to the need for regular blood level monitoring. Consequently, both clinicians and patients may have been hesitant to initiate or continue lithium treatment due to the restrictions imposed by the COVID‐19 pandemic.

The findings of this study should be interpreted with consideration of its limitations. Firstly, no information was available regarding the patients' diagnoses. As a result, we were unable to determine whether the different medications were used specifically for bipolar disorder or for other conditions. However, it is important to note that in the Netherlands, lithium is registered exclusively for the treatment of manic and depressive episodes associated with bipolar disorder in children, and for bipolar disorder or recurrent unipolar depressive episodes in combination with an antidepressant in adults. In addition, while the IADB.nl database records all prescriptions dispensed by community pharmacies, it does not verify whether the patients actually used the medications. Hence, in reality, actual lithium use may be even lower than reported in this study. Another limitation concerns the definition for new users that was used during this research. A new user was classified as an individual with no lithium dispensed for a 365‐day period prior to starting treatment. This definition may have resulted in episodic lithium users being misclassified as new users, which could lead to an overestimation of incidence rates. Furthermore, the exact ages of individuals in the IADB.nl database are not provided, as the database only records dates of birth on January 1st or July 1st of the corresponding year. Consequently, the actual age of the patient could exceed 25 by several months. To address this, only individuals up to the calculated age of 24.5 years were included, ensuring compliance to the age range of 6–25 years. Lastly, the duration of use may have been underestimated, and incidence rates potentially overestimated due to the absence of information on dispensing during hospitalization and in‐hospital medication use. Acute mania is typically diagnosed and managed in hospital settings, and the first outpatient prescription postdischarge would be captured by the IADB.nl database. This discrepancy could lead to inaccurate yearly incidence estimates, with new users being recorded later than their actual initial use.

## 5 Conclusion

The prevalence of lithium use in Dutch youth slightly increased between 2011 and 2022. However, the overall prevalence rates are still relatively low, considering that lithium is the first‐line treatment for maintenance therapy in bipolar disorder in the Netherlands. Our findings indicate that in the Netherlands, bipolar disorder is rarely diagnosed in children under the age of 12, as evidenced by the low prevalence rates observed in this age group. Concerns regarding lithium's potential side effects, the need for frequent monitoring, and the misdiagnosis of bipolar disorder may collectively contribute to prolonged periods of untreated illness and delays in the initiation of lithium therapy. More than half of the patients are initially dispensed SGAs, antidepressants or psychostimulants before lithium is introduced. These findings underscore the need for improved diagnostic accuracy in pediatric patients, adolescents and young adults, alongside further research into the safety of lithium use in younger age groups. Enhancing clinician education on the early recognition of bipolar disorder, considering lithium responders' profiles, and addressing barriers to lithium initiation by providing expert consultation may help optimize earlier and more effective interventions, ultimately improving clinical outcomes for individuals with bipolar disorder.

## Author Contributions

R.N.G., L.T.R., and B.D. designed the study. R.N.G., L.T.R., and C.C.M.S.‐V made a substantial contribution to finalizing the study protocol. J.H.J.B. collected and retrieved data from IADB.nl. R.N.G. and L.T.R. finished the data analysis. L.T.R. and R.N.G. wrote the first draft of the manuscript. B.D., B.C.P.K., C.C.M.S.‐V, E.H., J.H.J.B., M.H.J.H., and P.G.‐G. provided important comments on the draft manuscript. All authors read and approved the final version submitted for publication. The corresponding authors had full access to all the data in the study and had final responsibility for the decision to submit for publication.

## Funding

Lisa T. Ringeling, Bram Dierckx and Birgit C. P. Koch received grant research support from Erasmus Medical Center, Stichting de Merel and The Netherlands Organization for Health Research and Development (ZonMw), grant 10140022010011. Ravish N. Gangapersad received grant research support from Erasmus Medical Center and Smarter Choices for Better Health. Manon H.J. Hillegers received grant research support from the Dutch Research Counsel (NWO), grant 606360098021. The grant research supports had no further role in study design; in the collection, analysis, and interpretation of data; in the writing of the report; and in the decision to submit the article for publication.

## Ethics Statement

The study database IADB.nl comprises the de‐identified records and data collected in accordance with the national and European guidelines on privacy requirements (GDPR) for handling human data. Therefore, approval of the medical ethics committee was not required.

## Consent

The authors have nothing to report.

## Conflicts of Interest

The authors declare no conflicts of interest.

## Data Availability

The data that support the findings of this study are available in the database IADB.nl. However, these data are only made available after approval of a study protocol. Code Availability: Syntaxes used to analyze the data can be made available upon reasonable request.
